# Bronchoscopic balloon dilatation for tuberculosis-associated tracheal stenosis: a two case report and a literature review

**DOI:** 10.1186/s13019-016-0417-z

**Published:** 2016-01-29

**Authors:** Yong Fang, Xiaofang You, Wei Sha, Heping Xiao

**Affiliations:** Tuberculosis Center for Diagnosis and Treatment, Shanghai Pulmonary Hospital, Tongji University School of Medicine, Shanghai Key Lab of Tuberculosis, No.507 Zhengmin Road, Yangpu District, Shanghai, 200433 China

**Keywords:** Tracheal stenosis, Balloon dilatation, Tuberculous, Interventional therapy, Bronchoscopy

## Abstract

**Background:**

Bronchoscopic balloon dilatation (BBD) is a common strategy in the treatment of bronchostenosis. However, the longer dilating time in each inflation cycle (approximately 3–5 min) without mechanical ventilation is not possible for the treatment of tracheal stenosis.

**Case presentation:**

In this study, we reported our experience of BBD with shorter dilating time (10 s or 1 min) and intermittent ventilation for the repair of tuberculous-associated tracheal stenosis in two cases of our hospital. After the surgeries, the physical examinations and pulmonary function were tested. In case 1, the cough and dyspnea syndromes subsided, wheeze and strid or in lungs were remarkably reduced, tracheal lumen was considerably expanded and pulmonary function was improved following the treatment. For the case 2, her chest tightness, shortness of breath symptoms were alleviated after the treatment. The middle and lower trachea stenosis was dilated and patent, but the right main bronchus stenosis was slightly improved. No restenosis occurred in the two patients in 1 year outpatient follow-up.

**Conclusions:**

These findings suggest that our modification in BBD is safe and effective for treating this patient with tracheal stenosis caused by tuberculosis, but the longer-term effect of the surgery in a large number of patients with longer follow-up remains to be seen.

**Electronic supplementary material:**

The online version of this article (doi:10.1186/s13019-016-0417-z) contains supplementary material, which is available to authorized users.

## Background

Benign tracheal stenosis is a complication of various diseases and interventional managements [1–3]. Tuberculosis (TB) and endotracheal intubation are two most common causes of the tracheal stenosis. The symptoms of tracheal stenosis vary according to the site of the lesion and severity of tracheal narrowing. They usually include shortness of breath, cough, expectoration, stridor or, wheeze, and respiratory tract infections. If left untreated, tracheal stenosis might even lead to suffocation or tracheal cancer.

There are a variety of treatment options for patients with tracheal stenosis, which include surgical resection, balloon dilatation, stent placement, cryotherapy and laser therapy [4, 5]. Of the options, surgical resection and reconstruction are the best definitive treatment against tracheal stenosis [6]. However, many patients could not tolerate the surgery or are not suitable because of poor health condition, impaired pulmonary functions, anatomic limitations or technical difficulties. Stent placement is also a widely-used approach, but it might cause various complications, such as benign airway restenosis as a result of stent migration and granulation tissue proliferation [7].

In recent years interventional pulmonology technologies have been substantially advanced, and bronchoscopic balloon dilatation (BBD) characterized with lower morbidity and mortality has been increasingly recognized as an effective therapy for benign bronchus and lobe bronchus stenosis [8, 9]. However, there are few studies specifically targeting tracheal stenosis. Thus, this study aimed to evaluate the therapeutic effects of BBD on tracheal stenosis caused by TB in two cases. Moreover the longer dilating time in each inflation cycle (approximately 3–5 min) without mechanical ventilation during bronchus dilatation is not possible for the repair of tracheal stenosis. In this study, we designed shorter dilating time (10 s or 1 min) and intermittent ventilation for the repair of tracheal stenosis caused by TB (Additional file 1).

## Case Presentation

### Case 1

All persons have given their informed consent prior to their inclusion in the study, and all human studies have been approved by the local Ethics Committee and performed in accordance with the ethical standards.

A 20-year-old woman was admitted to her local hospital in June, 2010 because of cough and expectoration for 3 months. She was diagnosed as tracheal tuberculosis according to the positive acid-fast bacilli in her sputum and granuloma-like lesions on the tracheal wall with caseous necrosis by bronchoscopy with biopsy. She was given anti-TB medications consisting of Isoniazid (INH), Rifampicin (RFP), Ethambutol (EMB) and Pyrazinamide (PZA). However, her symptoms were not relieved, but deteriorated after 1 month. The bronchoscopy was performed again, revealing tracheal scar stenosis. The biopsy showed chronic inflammation of the upper tracheal mucosa with fibrous tissue proliferation. She was admitted to our hospital on December 9, 2010 for further treatment.

**Fig. 1:**
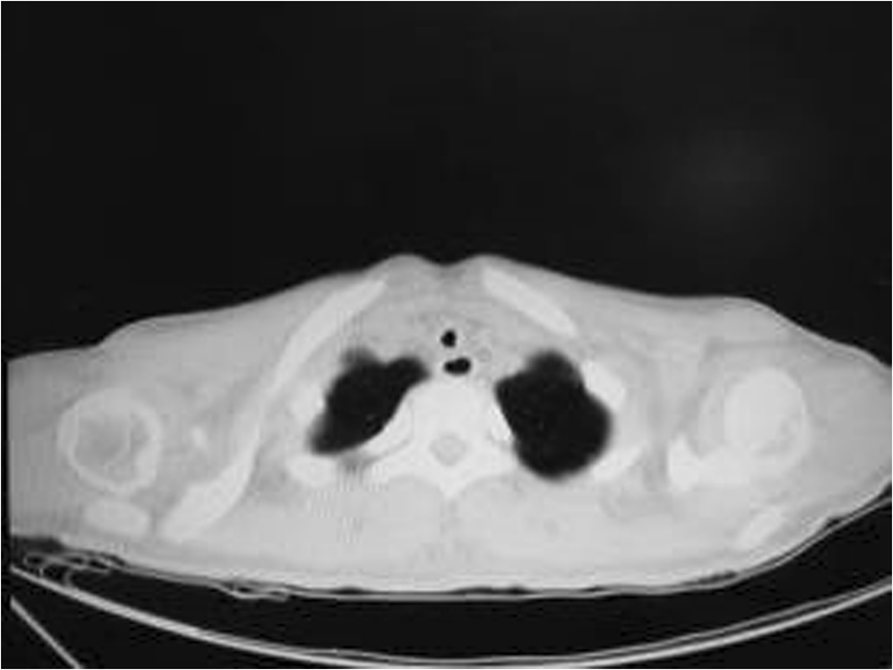
A chest computed tomography scan showing tracheal stenosis at thoracic inlet before treatment in Case 1

On admission, physical examinations showed that she was clear-minded, breath was 25 times per min, pulse was 93 beats per min, and blood pressure was 126/78 mmHg. On chest examination, her breath sound was rough with wheeze on both sides. The result of abdominal examination was normal. Pulmonary function testing (PFT) showed that her pulmonary function was impaired [vital capacity (VC) =3.29 L (91.9 % pred), the forced expired volume in 1 s (FEV1) =1.11 L (35.2 % pred), the ratio between FEV1 and the forced vital capacity (FEV1/FVC) =33.65 %]. A chest computed tomography (CT; Fig. 1) revealed tracheal stenosis at the thoracic inlet, patchy, nodule-like and streaking lesions with uneven density in both lobes of the lung, and normal lymph node sat mediastinum and lung hilus.

After admission, she continued to receive anti-TB drugs (INH, RFP, EMB and PZA), and then changed to anti-TB drugs [INH, RFP, EMB and streptomycin(SM)] due to liver injury. Electronic bronchoscopy was performed at 3 days after admission (December, 13) under general anesthesia. Mechanical ventilation was used to assist her breathing. The bronchoscopy showed a ring-like scar stenosis about 2 cm below the normal glottis. The bronchoscope (BF BC-260, Olympus Corporation, Tokyo, Japan) with an external diameter of4.9 mmcould pass through the stenosis, but not the bronchoscope (BF IT-260, Olympus Corporation, Tokyo, Japan) with the outer diameter of 5.9 mm. The bronchoscopy (BF BC-260) revealed that the airway wall was rough, the narrowed airway was about 5 cm in length, and the lower end of the stenosis was about 5 cm away from the sharp tracheal carina. No abnormality in the bilateral lobar bronchus was observed.

**Fig. 2:**
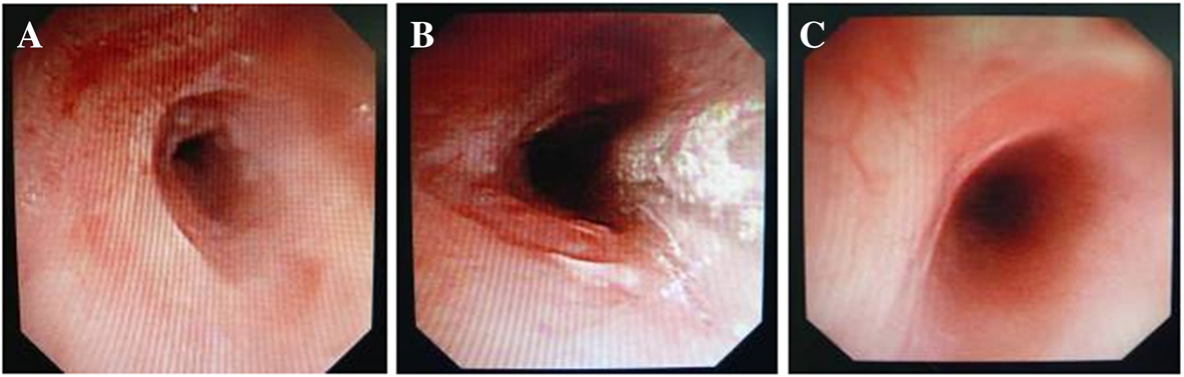
Photographs showing bronchus stenosis before and after treatment in Case 1. **a** Photographs showing bronchus lumen before treatment; **b** Photographs showing bronchus lumen immediately after treatment; **c** Photographs showing bronchus lumen 50 days after treatment

The tracheal stenosis was treated using the MK58880balloon (1 cm in diameter, Boston Scientific Cork Company, Ireland) with the pressure increased to 303.9 Kpa (3 atm) to dilate the trachea for 10s during which the mechanical ventilation was stopped. Subsequently, the pressure started to decrease and the mechanical ventilation was recovered. The balloon dilatation was repeated 3 times. The bronchial lumen expanded only slightly following the procedure (Fig. 2a, b). On the second day of the balloon dilatation, her symptoms including coughing, shortness of breath, were alleviated. The wheeze and stridor of lungs were reduced under physical examination.

Ten days later, she received bronchoscopic re-examination of the airways and the results still indicated that the bronchoscope (BF BC-260, Olympus Corporation, Tokyo, Japan) could pass through the stenosis, but not the bronchoscope (BF IT-260, Olympus Corporation, Tokyo, Japan). Thus, the high-pressure balloon dilatation treatment was performed again during which the pressure of the balloon was increased to 5 atm and then maintained for 10 s. After 8 inflation cycles, the bronchoscope (BF IT-260, Olympus Corporation, Tokyo, Japan) can pass through the stenosis. However, her cough and shortness of breath symptoms were deteriorated following the treatment, which may be caused by the presence of bronchial edema and spasm. These symptoms were resolved by intravenous and intranasal administration of dexamethasone using atomization device. On the third day after treatment, her cough and shortness of breath symptoms were apparently ameliorated. The physical examinations showed that wheeze and stridor of lungs were remarkably reduced. Then, she was discharged.

Fifty days after the treatment, a repeat PFT showed that her pulmonary function was remarkably improved (VC = 3.40 L (94.8 % pred), FEV1 = 2.06 L (65.5 % pred), FEV1/FVC = 60.51 %). A repeat bronchoscopy showed that the ring-like scar stenosis was dilated, allowing the passage of bronchoscopy (BF IT-260, Olympus Corporation, Tokyo, Japan) (Fig. 2c). During her 1 year outpatient follow-up, no tracheal stenosis was observed. Timeline of past history of the patient was listed in Table 1.

**Table 1:** Timeline of case 1

Time	Important event
Dec 9, 2010	Admission
Dec 13, 2010	First balloon dilation treatment
Dec 23, 2010	Second balloon dilation treatment
Feb 12, 2011	Pulmonary function check-ups

### Case 2

A 39-year-old female patient was admitted to our hospital on October 13, 2010 because of cough and expectoration for 3 years, chest tightness and shortness of breath for approximately 1 month. On November, 2007, she was ever admitted to a local hospital with complaints of cough, expectoration and white phlegm, but not fatigue, night sweats, fever and chest pain. The local hospital diagnosis was bronchial asthma. She was given anti-inflammatory drugs, but her symptoms persisted.

**Fig. 3:**
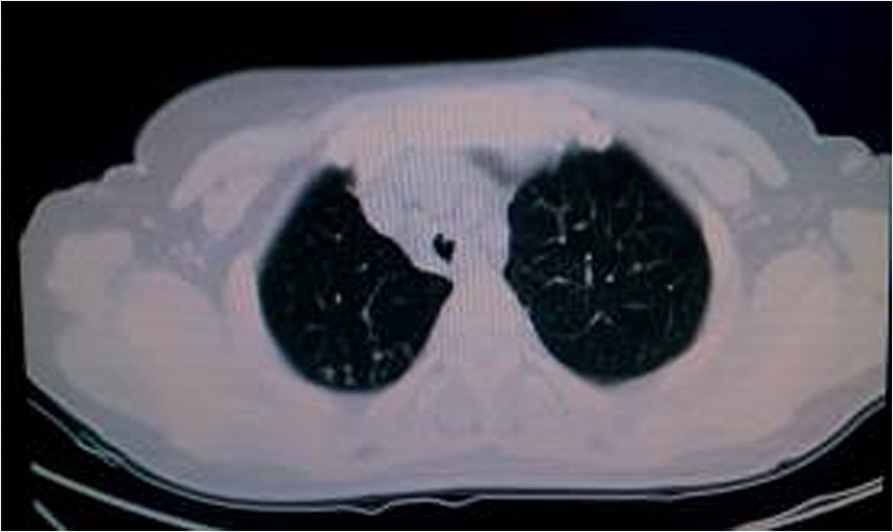
A chest computed tomography scan showing tracheal stenosis and right bronchial stenosis of the patient in Case 2

**Fig. 4:**
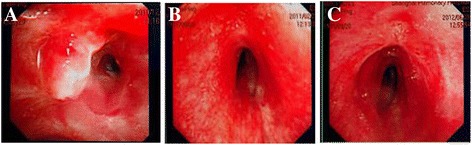
Photographs showing tracheal and bronchus stenosis in Case 2. **a** Photographs showing the middle and lower tracheal stenosis, and right main bronchus stenosis with caseous necrotic material attached to the nozzle exit before the treatment; **b** Photographs showing the narrowed trachea and right main bronchus immediately after the first treatment; **c** Photographs showing the middle and lower tracheal, and right main bronchus after the fifth treatment

On admission, physical examinations revealed that she was clear-minded, breath was 26 times per min, pulse was 100 beats per min, and blood pressure was 132/80 mmHg. Chest examination revealed wheeze in the trachea, and rough breath sound with in aspiratory and expiratory wheeze in both lungs. Her abdominal examination was normal. No pathological syndrome was observed. Chest CT scan revealed pulmonary TB on both sides, especially in the middle and lower lobe. Lower tracheal stenosis, right bronchus stenosis and right pleural thickening were also observed (Fig. 3). Sputum smear for acid-fast bacilli was positive. Subsequently she was given combined anti-TB treatment (INH, RFP, EMB and PZA) since December 13, 2010. On December 17, 2010, bronchoscopy was performed under local anesthesia, revealing that as shown in Fig. 4a, the glottis was normal, false diverticula was present in the middle segment of trachea, and stenosis was present in the middle and lower trachea, barely allowing the passage of the bronchoscopy (BF BC-260, Olympus Corporation, Tokyo, Japan). Tracheal mucosal congestion and swelling were present, and the left main bronchus and lobar bronchus on the left side was patent, although with the presence of viscous secretions. However, the right main bronchial stenosis was observed with caseous necrotic material attached to the bronchial orifice and could not permit the passage of the bronchoscopy (BF BC-260, Olympus Corporation, Tokyo, Japan). Our final diagnosis was active secondary pulmonary tuberculosis, tracheal and right main bronchial tuberculosis. On January 12, 2011, the patient was administrated with endotracheal intubation. After a month, the endotracheal catheter was removed and then, the patient was discharged.

On February18, 2011, she was re-admitted to our hospital. The bronchoscopic high-pressure (6 atm) balloon dilatation (MK58880, Boston Scientific Cork Company, Ireland) was performed in the middle and lower trachea for the patient under general anesthesia and electrocardiogram (ECG) monitoring. Mechanical ventilation was applied to assist the patient’s breathing. When the oxygen saturation was below 95 %, the balloon stopped dilatation and started empting. The balloon dilatation lasted as long as 1.5 min for each cycle. When the oxygen saturation recovered to be above 99 %, the second balloon dilatation was performed and repeated. The bronchoscopic high-pressure (5 atm) balloon dilatation was also performed under ECG monitoring for the right main bronchial stenosis twice with5 min for each cycle. After the balloon dilatation, the BC-260bronchoscope could pass through the middle and lower trachea stenosis but not the IT-260bronchoscope. For the right main bronchus stenosis, BC-260 bronchoscope could not even pass (Fig. 4b). Her chest tightness, shortness of breath symptoms were improved after the balloon dilatation treatment, but still could not tolerate PFT.

She received balloon dilatation treatment for 5 more times on March 28, April 22, May 6, June 7 and July 18, 2011, respectively. On June 28, 2012, a repeat bronchoscopy showed that the middle and lower trachea stenosis was dilated and patent without the presence of congestion and swelling, allowing the passage of the IT-260 bronchoscope. The orifice of the right main bronchus was still narrowed, could not permit the passage of the bronchoscopy (BC-260), but the caseous necrotic materials disappeared (Fig. 4c). No restenosis occurred in the patient in 1 year outpatient follow-up. Timeline of case 2 was listed in Table 2.

**Table 2:** Timeline of case 2

Time	Important event
Oct 13th, 2010	Admission
Dec 17, 2010	Bronchoscopy examination
Feb 18, 2011	First balloon dilation treatment
Mar 28, 2011	Second balloon dilation treatment
Apr 22, 2011	Third balloon dilation treatment
May 6, 2011	Fourth balloon dilation treatment
Jun 7, 2011	Fifth balloon dilation treatment
Jul 18, 2011	Sixth Pulmonary function check-ups

### Literature review

We searched the WANFANG and PubMed database for articles published between 1984 and 2014 in the Chinese-and English-language literature. There were several studies on the application of balloon dilatation in management of combination of main bronchus and lobe bronchus stenosis, yet rare reports on specific treatment of TB-associated tracheal stenosis by BBD were retrieved. The search of PubMed database yielded 13 articles. A total of 474 patients were involved in the retrieved articles from PubMed database and the present study. The total information retrieved was summarized in Table 3 [8–20]. Of the 474 patients, the tracheal stenosis of 4 infant patients results from endotracheal intubation [10]. The etiologies of the 474 patients include bronchial carcinoma, other malignancy, lung carcinoma post-tracheal resection, endotracheal intubation, tracheal tuberculosis, prolonged mechanical ventilation, lung transplantation, Wegener’s granulomatosis, idiopathic stenosis, post-tracheostomy, sarcoidosis and amyloidosis.

**Table 3:** Summary of clinical characteristics of the patients in the retrieved articles and the present study

Category		Number of cases
Total		476
Malignant stricture		85
	Bronchial carcinoma	78
	Magligance	7
Benign stricture		391
	Endotracheal intubation	84
	Tracheal tuberculosis	72
	Prolonged mechanical ventilation	11
	Lung transplantation	166
	Lung carcinoma	4
	Wegener’s granulomatosis	6
	Idiopathic stenosis	4
	Post-Tracheostomy	31
	Sarcoidosis	3
	Amyloidosis	2
	Unknown	1
Ventilation mode	Intermittent ventilation	7
	Ventilation ducts	13
dilation duration	10-30 s	256
	30s-1 min	3
	1-3 min	2
	40 min	1
Ballon pressure (atm)*	<3	3
	3-5	81
	>5	3
Outcome	Valid	176
	Invalid	25
Follow-up (months)	0-6	79
	6-12	2
	>12	395
Complication	Granulation tissue formation	30
	Tracheoesophegial fistula	1
	Tracheal laceration	4
	Bronchospasm	1
	Laceration of bronchial artery	1

## Discussion

BBD has become a preferred option in the management of tracheal and bronchial stenosis. The study assessed the effect of high pressure BBD in two patients with TB—associated tracheal stenosis. The tracheal lumen was dilated and pulmonary function was improved following the treatment.

There are usually two ventilation modes used in balloon dilatation treatment: intermittent ventilation and catheter ventilation. The catheter ventilation refers to inserting the ventilator-linked catheter into trachea, which permits easy breathing of the patients undergoing balloon dilatation treatment. However, it carries a variety of complications such as ventilator-associated pneumonia, pneumothorax and alveolar damage. With regard to intermittent ventilation, ventilation is stopped for the patient when the balloon is dilated, and the ventilation is recovered at intermission between balloon dilatations, thus reducing the risk of the complications. In the present study, intermittent ventilation was applied to the 2 cases.

In most cases, single balloon dilatation for bronchus stenosis usually lasts 3–5 min. The longest balloon dilatation duration is 40 min [9]. The longer dilating time in each inflation cycle without mechanical ventilation during bronchus dilatation is not possible for the treatment of tracheal stenosis. Thus, we introduced the shorter dilating time for treatment of case 1 (10 s) and case 2 (1.5 min) in reference to the recent studies of Shitrit et al. [8] and Low et al. [20].

In the 474 cases retrieved, balloon pressure varies according to the site and the type of the stenosis. The pressure for two infant patients is 4 atm. Notably, 16 atm is used in two cases, but induces tracheal wall laceration [14], indicating high pressure might be dangerous. However, a novel high-handed BBD method (14 atm) is used for a female patient with right middle segmental bronchostenosis. The procedure lasts for 40 min. After the procedure, her intermediate bronchus is observed again, the right lower and middle lobes are reopened [9].

Symptoms, lung functions and lumen diameter are primary indicators of the efficacy of the BBD treatment. However, tracheal stenosis is in uneven and irregular form, and measurement of stenosis diameter is usually based on researchers’ observation and captured images of tracheal stenosis. Therefore, the measurement result of stenosis diameter is inaccurate and unconvincing. In the present study, whether the same type of bronchoscope could pass through the narrowed lumen after the treatment was regarded as a direct indicator of the treatment outcome. Because the tracheal lumen usually recoils after the BBD procedure, long-term assessment for the effect of the treatment is considered more important than immediate assessment after the procedure. In a recent study, the effect of balloon dilatation on a female patient with bronchial TB-associated bronchostenosis is assessed following 13 months of anti-bronchial TB treatment. Dilatation of the bronchostenosis is observed, and no complication occurs in the patient [9]. In consistence with the finding, the effect of BBD was assessed for two cases in 1 year outpatient follow-up after the treatment in the present study.

Although patients often show immediate satisfactory result following BBD treatment, the effect fails to last long. Long term follow-up reveals that additional treatments, such as laser treatment and stent therapies are required by most patients after the BBD treatment [8]. It has been reported that syndromes of 21 consecutive patients with broncheal stenosis are immediately relieved following bronchoscopic interventions. However, out of the 21 patients, only 11 remain asymptomatic at the end of 25 months follow-up [20]. Besides, repeat sessions of bronchoscopic interventions are required by patients [20]. These studies unveil that bronchoscopic interventions are palliative measures in the management of airway stenosis.

Primary complications of the balloon dilatation treatment usually include pneumothorax, mediastinal emphysema, tracheal and broncheal wall laceration, and hemoptysis. Broncheal wall laceration occurs in two cases, due to the exceedingly high pressure (up to 16 atm) [14]. Therefore, the pressure of balloon inflation should be in tight control during the procedure. On the same day of the BBD treatment, the two patients presented with symptoms of cough and dyspnea in the current study. The symptoms were deteriorated when the balloon was dilated, and then relieved by subsequent intravenous administration and atomizing inhalation of dexamethasone. One possible explanation for the symptoms was the presence of local congestive edema and airway spasm caused by the high pressure balloon dilatation. The congestion and edema would be exacerbated by the increased pressure. In order to prevent these complications, necessary preparations must be made prior to and post-surgery. Once the symptoms are present, immediate measures should be taken to alleviate the syndromes in patients. Despite these complications, the surgery is considered safe since it is performed under strict monitoring.

Furthermore, considerable efforts have been made to improve the effect of BBD and to avoid severe complications. For instance, multi-modality treatment consisting of electrocautery, balloon bronchoplasty, and subsequent topical application of mitomycin-C has been used to treat a patient with benign tracheobronchial stenosis. The stenosis is dilated, no re-stenosis or complication is observed following the treatment [19]. Electrocautery is used to cut open the fibrotic stricture prior to balloon dilatation, thus high pressure is not necessary to dilate the balloon. Consequently, the complications caused by excessive high pressure could be avoided. Moreover, flexible bronchoscopy therapeutic modalities consisting of balloon dilatation, laser resection, high-dose rate (HDR) endobronchial brachytherapy and self-expanding metal stent placement have used in elderly patients with granulation tissue formation because of refractory stent. The HDR endobronchial brachy-therapy is widely-used to overcome mechanical obstruction of major airways, and could prevent and treat stent-related granulation tissue formation. The success rate of the flexible bronchoscope-based morbidities is up to 87 % [17]. Furthermore, BBD has been proved to be safe and effective in the management of bronchial stenosis following lung transplantation. It facilitates easy stent placement and reduces the use of stent placement in patients, thus decreasing the complications induced by stent placement [18].

## Conclusions

The bronchoscopic high-pressure balloon dilatation was a safe and operable method for treating patients with tracheal stenosis caused by TB. There were only two cases described in the study. The longer-term effect of the surgery in a large number of patients with longer follow-up remained to be seen.

## Consent

Written informed consent was obtained from the patient for publication of this Case report and any accompanying images. A copy of the written consent is available for review by the Editor of this journal.

## Additional file

Additional file 1:
**CARE Checklist (2013) of information to include when writing a case report.** (DOCX 1493 kb)

## Abbreviations

BBD: Bronchoscopic balloon dilatation
TB: Tuberculosis
INH: Isoniazid
RFP: Rifampicin
EMB: Ethambutol
PZA: Pyrazinamide
PFT: Pulmonary function testing
